# A situational picture of HIV/AIDS and injection drug use in Vinnitsya, Ukraine

**DOI:** 10.1186/1477-7517-2-16

**Published:** 2005-09-15

**Authors:** Katerina Barcal, Joseph E Schumacher, Kostyantyn Dumchev, Larisa Vasiliyevna Moroz

**Affiliations:** 1 Department of Epidemiology, School of Public Health, The University of Alabama at Birmingham, Birmingham, Alabama, USA; 2 Division of Preventive Medicine, Department of Medicine, The University of Alabama School of Medicine, Birmingham, Alabama, USA; 3 Department of Infectious Diseases and Epidemiology, Vinnitsya National Pirogov Memorial Medical University, Vinnitsya, Ukraine; 4 Vinnitsya Regional Narcological Dispensary, Vinnitsya, Ukraine

**Keywords:** HIV/AIDS, IDU, drug abuse, Ukraine, Rapid Assessment and Response Guide

## Abstract

**Background:**

New and explosive HIV epidemics are being witnessed in certain countries of Eastern Europe, including Ukraine, as well as a rapid and dramatic increase in the supply, use, and negative public health consequences of illicit drugs. A majority of registered HIV cases in Ukraine occur among injection drug users (IDUs), large numbers of whom report HIV risk behaviors such as needle sharing. The purpose of this study was to apply the World Health Organization's Rapid Assessment and Response on Injection Drug Use (IDU-RAR) guide to create a situational picture in the Vinnitsya Oblast, Ukraine, a region with very scarce information about the HIV/AIDS and injection drug use (IDU) epidemics.

**Methods:**

The IDU-RAR uses a combination of qualitative data collection techniques commonly employed in social science and evaluation research to quickly depict the extent and nature of the given health problem and propose locally relevant recommendations for improvement. The investigators focused their assessment on the contextual factors, drug use, and intervention and policy components of the IDU-RAR. A combination of network and block sampling techniques was used. Data collection methods included direct observation, review of existing data, structured and unstructured interviews, and focus group discussions. Key informants and locations were visited until no new information was being generated.

**Results:**

The number of registered HIV cases in Vinnitsya has increased from 3 (1987–1995) to 860 (1999–10/2004), 57 of whom have already died. Ten percent of annual admissions to the area's Regional Narcological Dispensary were for opiate disorders, and the number of registered IDUs rose by 20% from 1999 to 2000. The level of HIV/AIDS awareness is generally poor among the general population but high among high-risk populations. Both HIV/AIDS and injection drug use carry a strong stigma in the community, even among medical professionals. There was very little evidence of primary HIV/AIDS prevention efforts, and IDU prevention efforts focused on promotion of anti-drug messages in the schools.

**Conclusion:**

Given that Ukraine has sparse resources to be devoted to this problem, action recommendations should be prioritized, realistic, and initially targeted to persons in greatest need. The following action recommendations are prioritized by the following categories: First priority: Voluntary Counseling and Testing; Second Priority: Prevention and Education; and Third Priority: Harm Reduction and Treatment. They are provided in this sequence based on what response can realistically be implemented first with limited additional resources and can make the greatest immediate impact. The persons at greatest risk, HIV positive persons and IDUs, should be attended to first.

## Background

New and explosive HIV epidemics are being witnessed in certain countries of Eastern Europe: Russia, Moldova, Belarus, and Ukraine [1-6]. According to the Ukrainian Centre for AIDS Prevention, a cumulative total of 71,359 HIV cases and 4,851 deaths were registered in Ukraine by October, 2004 [11]. The actual prevalence is believed to be much higher, with the true number of existing cases estimated at a staggering 180,000–590,000. Such a figure would make Ukraine the most affected country in the region [7].

Political independence in Ukraine and surrounding Eastern European countries in the 1990s has been associated with a rapid and dramatic increase in the supply, use, and negative public health consequences of illicit drugs [8-10,16]. While little reliable epidemiological data on opiate injection drug use (IDU) is available from Ukraine, existing data defines it as significant concern. There were 83,868 officially registered cases of drug addiction in Ukraine by the end of 2002 [23]. The existing registration system does not distinguish IDU from other types of drug use, but one author suggests that 96% of all drug dependent registered patients injected drugs [12]. According to experts' opinions, the existing number of injection drug users (IDUs) exceeds official registry data by a factor of 5 to 7 times, depending on the particular region [13].

The majority of reported HIV infections in Ukraine were diagnosed in IDUs from 1987 to 2004 [14,15]. A total of 40,809 IDU-related HIV cases in Ukraine were reported from 1987 to 2004, which comprises 71.7% of all adult cases [11]. These figures represent only registered cases and therefore underestimate the number of diagnosed cases. An estimated 180,000 to 590,000 persons in Ukraine are infected with HIV, with IDU the primary source of transmission [7]. HIV risk behaviors such as needle sharing at the time of last injection were reported by 47% of IDUs [17], and only 29% of IDUs have reported consistent use of clean needles [13].

The proportion of total cases acquiring HIV through opiate IDU in certain areas of Ukraine dropped from 72.7% in 1997 to 56.7% in 2004, suggesting expansion of the epidemic to the general population [6-11]. Data indicate that an HIV epidemic fuelled by heterosexual transmission is emerging, and its expansion will depend on the size of bridge populations that link high-risk groups with the general population [4]. In comparison, of all diagnosed AIDS cases as of December 2000 in the United States, 25% occurred among IDUs [18]. Although the extent of illicit drug use is probably more limited than the extent of many other social problems in the countries of the former Soviet Union, the extreme growth and relevance to HIV/AIDS make it a primary area of concern to more than 200 million people living there [8].

## Purpose

At the time of this study, information about the HIV epidemic came mainly from the most heavily affected Ukrainian cities, such as Odessa, Kharkiv, and Mykolaiv [1]. The attempts in these and several other cities to control the epidemic were documented in the areas of youth-based HIV education and social marketing, harm reduction programs (such as needle exchange), and narcological hospitals. However, there was very little information about the HIV/IDU situation in smaller cities and rural areas of the country. It was not clear how the epidemic progressed in other parts of the country and whether any prevention or treatment efforts existed outside of the major cities. The purpose of this study was to create a situational picture of the injection drug use and HIV/AIDS and IDU epidemics in the Vinnitsya Oblast (a semi-urban community in central Ukraine with scarce information about the nature and extent of the epidemics) and propose action recommendations for positive change in education, service, and research.

This project builds on a successful three-year joint collaboration between public health, HIV/AIDS, and drug addiction researchers and clinicians from the University of Alabama at Birmingham (UAB), The Regional Narcological Dispensary (RND) in Vinnitsya, Ukraine (a local government-run alcohol and drug addiction hospital), and the Vinnitsya National Pirogov Medical University (PMU). This collaboration was initiated by the UAB John J. Sparkman Center for International Public Health Education and an International Clinical, Operational and Health Services Research and Training Award (ICOHRTA) awarded by the National Institutes of Health Fogarty International Center to UAB in 2001.

The fieldwork phase of this study was completed from May through July of 2001.

## Methods

This study utilized rapid assessment and response (RAR) methodology developed by the World Health Organization (WHO) in 1998. The RAR method uses a combination of qualitative data collection techniques commonly employed in social science and evaluation research to quickly depict the extent and nature of the given health problem and propose locally relevant recommendations for improvement. One of the key principles of RAR is that data are collected from different sources, which allows continuous examination of the reliability and consistency of the data and enables investigators to make better informed decisions about what evidence should be sought in the next stage of the assessment. RAR is designed to rapidly assess a current problem situation (e.g. IDU) in a community. This information is then used to make informed decisions about the development of interventions needed to reduce the adverse health and social consequences of the targeted condition. The WHO Rapid Assessment and Response guide on injection drug use (IDU-RAR) [19] was deemed appropriate for this study because it has been used successfully in resource-limited settings within the United States and around the world [20], including two Ukrainian cities – Odessa [1] and Kharkiv [13].

Due to limited financial resources and a short time frame, we directed our focus to three areas of the IDU-RAR when assessing the IDU situation: Contextual Assessment, Drug Use Assessment, and Intervention and Policy Assessment. The Contextual Assessment identifies factors that influence the current and potential situations regarding drug injection and its adverse health consequences, as well as opportunities for the development of interventions. Key areas for assessment include factors for spread of IDU, exacerbation versus amelioration of adverse health consequences of injecting, and factors that are likely to hinder or enable the development of interventions. The Drug Use Assessment focuses on the nature and extent of drug use – who is injecting drugs and where this occurs, as well as trends in injection drug use over time. Finally, the Intervention and Policy Assessment is designed to assess existing interventions and policy responses aimed at reducing drug use and its consequences while allowing the assessment team to examine their effectiveness and develop recommendations. Our study also included an assessment of the HIV/AIDS situation, which incorporated the nature and extent of the epidemic, HIV/AIDS awareness, attitudes toward individuals living with HIV/AIDS, and existing prevention/control measures.

### Mapping of the Vinnitsya Community

As a first step of the rapid assessment, the principal investigator met with key collaborators in Vinnitsya to assemble an assessment team and identify key informants. Immediately following the initial consultation, a conceptual map of the Vinnitsya community was developed. The map captured key locations for the needs assessment including major gathering points for IDUs, areas where drugs are sold, needle exchange sites, treatment facilities, transportation routes, and other key locations. The investigator traveled to all key locations to develop a physical map, noting activities that would help to provide some insight into the nature of IDU.

### Sampling

In order to create a representative sample of informants, a combination of *network *and *block *sampling techniques was used. The network sampling method involved a chain of referrals initiated by the key informants. All key informants were asked to provide a list of individuals who would be able to offer additional information on a given area of the needs assessment. These individuals were then contacted for further information and asked to provide another list of informants. This approach was used until no new information was being generated. The block sampling technique was similar to the networking sampling, but instead of using key informants as the starting point, the needs assessment team traveled to key locations (identified by the mapping exercise) to identify new informants. As an example, the leading investigator frequently traveled to a local market in the Vishinka district (identified as a gathering point for IDUs) to develop rapport with IDUs and gain access to new informants who would be willing to provide information on the IDU situation in Vinnitsya.

### Rapid Assessment Implementation

Data were collected using a combination of rapid assessment methods, including direct observation, review of existing data (including statistical data from government reports, annual reports from non-governmental organizations [NGOs], local research studies, and media), structured and unstructured interviews, and focus group discussions. An important component of the needs assessment was the triangulation of information, or cross-checking of collected data through the multiple sources. This allowed for collection of more representative data with higher confidence in its accuracy.

## Results

### Extent of the HIV/AIDS epidemic

The first occurrences of HIV infection in the Vinnitsya region were officially registered with the Sanitation and Epidemiological Service between 1987 and 1995. At this point, only 3 HIV infections were registered. By 1996, the number of new HIV infections rose to 46 (for a total of 49 HIV infections), including the first officially registered AIDS case. Between 1999 and 2001, there was a sharp increase in the number of new HIV infections in the region (at approximately 130%) that brought the total number of HIV infections to 431. At the end of 2001, a total of 112 new HIV infections, 26 AIDS cases, and 17 AIDS-related deaths were recorded for the Vinnitsya region. The regional Sanitation and Epidemiological Service statistics for the year 2001 revealed that the largest proportion of HIV infections occurred through injection drug use (83.9% of the total), followed by sexual transmission (16.1% of the total). Individuals in the 20–29 age group accounted for the largest proportion of HIV infections (69.6 %). Within this age group, the male to female ratio was 3 to 1. The most heavily affected areas within the Vinnitsya Oblast were Gaisyn, Ladyzhyn, and the city of Vinnitsya with 29.5%, 20.5%, 15.1% of the total number of new HIV infections in 2001 respectively. By October 2004, there were 783 cases of HIV infection and 57 AIDS deaths registered in Vinnitsya Oblast. In 2004 for the first time heterosexual route of transmission has prevailed with 54.1% of newly registered cases [24].

Information gathered from NGOs and local researchers suggests similar trends. However, accurate prevalence rates at the present time are difficult to assess due to lack of testing resources and fear and mistrust among those who may be at risk of having HIV/AIDS.

### HIV/AIDS awareness

Data collected through personal interviews with key informants and focus group discussions suggest that residents of Vinnitsya have heard of HIV/AIDS, but the level of knowledge and perception of risk vary among different groups. A focus group discussion with students from local universities in Vinnitsya revealed that young people in the community were likely to perceive HIV/AIDS as something that is found exclusively in the West and thus is not a real threat to their community. Many of the students were surprised to learn that HIV cases were reported in Vinnitsya and admitted that they were not as well informed about the disease as they needed to be. Discussions with IDU at a needle exchange program in the Vishenka District, on the other hand, revealed a much different outlook on the HIV/AIDS situation. These individuals were well aware of the presence of HIV in the community, and some even knew someone who had already contracted the virus. They felt that they were well informed about HIV/AIDS and how to protect themselves against the virus. Interviews with Vinnitsya city service providers and representatives from local NGOs confirmed that the level of HIV/AIDS awareness among high-risk populations was high. The same level of HIV/AIDS awareness, however, did not appear to extend to the rural communities. According to information from rural health providers working with IDUs, many of their patients lacked information about HIV/AIDS and did not perceive themselves as being at risk. A director of a government program focusing on the social welfare of youth in the Vinnitsya region also attested to low HIV/AIDS awareness among young adults in the rural communities.

### Attitudes toward individuals living with HIV

Vinnitsya residents living with HIV face a great deal of stigma and lack a widespread support system within their community. According to infectious disease treatment providers at a local hospital in Vinnitsya, HIV positive patients tend to stay secluded and often are very concerned that other people not learn about their infection. It is not unlikely for someone diagnosed with HIV infection to seek treatment and consultation with a physician during evening hours or at other times when they would be less likely to be seen by other people at the medical facility. Though many physicians seemed compassionate toward HIV positive patients (particularly infectious disease specialists working with HIV patients), direct observations and interviews with medical staff revealed that HIV positive individuals were stigmatized within treatment facilities. Test results that were to be kept confidential were openly shared in patient charts, and HIV patients were placed in isolation blocks. Interviews with nurses and other clinical staff revealed that some of the staff were reluctant to treat HIV infected patients. HIV positive patients at a local narcological dispensary stayed in an isolation block consisting of a small single-bed patient room, a nurse's cabinet, and a small waiting area. Anyone in the facility could easily determine who was staying in the isolation block and why he or she was there (as the restricted area was clearly labeled). It was not until recently (at the time of writing this paper) that the isolation block was discontinued and persons diagnosed with HIV/AIDS mainstreamed into the hospital.

Aside from counseling provided by some physicians who work with HIV-positive patients, there was very little evidence of routine counseling and follow-up for individuals who are tested for HIV. An important concern expressed by a physician at a hospital-based HIV/AIDS center was the lack of a support system that would allow patients diagnosed with HIV to speak openly about their condition and gain more information on living with the virus.

### HIV/AIDS prevention

Prevention activities found in the area were classified according to the three categories: primary (prevention of occurrence), secondary (diagnostics and treatment), and tertiary (minimization of adverse consequences). With the exception of a school-based educational program and some media efforts, there was very little evidence of primary HIV/AIDS prevention. The school-based program, organized by a local NGO, was implemented at only one of the 40 local elementary schools. The program (modeled after the American Project Hope) utilized innovative educational methods, through which children took part in skill building exercises (including self-efficacy building) and learned through peer-education. This program, however, did not have a built-in evaluation or measures to ensure sustainability. The local media were known to disseminate HIV/AIDS information through newspaper articles, but none of the information concentrated on the prevention of HIV/AIDS. At the time of the study, Peace Corps volunteers based in Vinnitsya were planning to develop and implement an HIV/AIDS education program through UNAIDS-Kyiv, which would serve as a great opportunity to create more educational programs at local schools in Vinnitsya and surrounding rural areas.

Primary prevention efforts carried out by a local NGOs reached out to high-risk populations, including commercial sex workers and IDUs, in the city of Vinnitsya and surrounding smaller cities. Outreach workers at needle/syringe exchange sites distributed condoms and information about HIV/AIDS and other sexually transmitted diseases. Educational booklets included information on safer sex practices and where to go for treatment. IDUs participating in the needle-exchange program were referred to a local hospital that had a special hepatitis and HIV/AIDS center within its facility. Some referrals to substance abuse treatment also were made. The program provided opportunities for HIV, HBV, and HCV testing free of charge. This effort, however, was fully funded through a time-bound grant, and it was not clear whether it would be sustained.

The AIDS Center at the local hospital provided HIV infected individuals with necessary medical attention and some counseling services and linked them with other sources of information. There were limited resources, however, for secondary prevention activities, such as screening, analysis, and HIV/AIDS disease monitoring. Availability of antiretroviral therapy was limited due to high cost. Patients had to make arrangements to buy their own medications. This situation was likely to change with the Ukrainian government's intention to provide subsidized antiretroviral therapy in the near future, supported by the grant from the Global Fund. The AIDS Center also worked closely with local NGOs to recruit individuals from high-risk populations for testing and treatment.

### Injection drug use

During the Soviet era, IDU was largely hidden and mostly limited to individuals from wealthy families. The drugs that were consumed during that time usually included "clean" drugs such as morphine and heroin. Substance abuse treatment providers who treated patients during the Soviet era indicated that the nature of drug use in Vinnitsya has changed dramatically over the past decade. In the years following Ukraine's independence, drug use has increased rapidly and shifted from purer, more expensive drugs to drugs that can be made at home. According to recovering IDUs from a local church, raw materials became easily accessible, and preparation procedures were passed around like cooking recipes. One especially potent amphetamine-like injectible drug, commonly referred to as "vint," was prepared using readily available chemicals that could be purchased at drug stores and local pharmacies. Though this particular recipe came at a price, IDUs could easily learn how to prepare a homemade opiate solution ("hanka" or "shirka") from their peers.

Individuals who were new to IDU could purchase ready-filled syringes or small medicine containers with the opiate solution at a local market. According to informants at a market in the Vishinka District of Vinnitsya city, drug dealers (often IDUs themselves) were able to do their business there without getting much attention from law enforcement while having good access to new customers. An inexperienced person might be invited to the dealer's apartment or a nearby garage, as attested by social workers at a local needle exchange program, where the dealer would help him or her to inject the drug.

There were no reliable epidemiological studies available to estimate the prevalence rates of opiate IDU in Vinnitsya and surrounding areas. Ten percent of the annual admissions to the area's only RND for drug and alcohol treatment were diagnosed with opiate disorders, but the actual prevalence rate in Vinnitsya was estimated to be more than ten times that figure or 2,000 persons with opiate disorders (personal communication with Pavel Slobodyenyuk, M.D, 2004). Dr. Slobodyenyuk also reported that the number of registered IDUs from rural areas during the year 2000 rose by 20% from the previous year. According to reports from the regional Sanitation and Epidemiology Service, rural areas were the most heavily affected in terms of opiate IDU and reportedly include the regions of Koziatyn, Zhmerynka, Trostyanets, Ladyzhyn and Illinitsi, as well as various small towns such as Hnivan and Vapniarka.

### Factors that encourage the spread of IDU

Results from this study indicated a combination of numerous factors that encouraged the spread of opiate IDU, including Ukraine's economic situation, social changes following the country's independence, easy access to poppy plants, and lack of knowledge. As Ukraine transitioned from the old (i.e. Soviet) system, many people were left without jobs or ones that pay on average US $30–50 a month as revealed by community informants. The sale of *hanka *quickly became a prosperous business. The drug production was not difficult, the raw material was grown in the area, and the demand for opiates was increasing. The cultivation of poppies has been part of the Ukrainian culture for many generations. Consequently, there was fairly easy access to poppies in the Vinnitsya region, especially in the rural areas. IDUs were able to gather poppy straws directly from the fields or purchase them from *babushkas *(grannies) at the local market. A 1-ml dose of *hanka *sold for about 5.00 grivnas (about $0.95 US) during the poppy season and for about 8.00 grivnas (about $1.50 US) out of season according to local IDUs. Selling a liter of *hanka *in a day allowed one to make more than ten times the income an average person makes in a month.

There did not appear to be a single driving force behind the increase in drug use among young adults. Discussions with IDUs at local treatment facilities and needle exchange programs revealed that people turn to drugs for different reasons. Some people sold drugs to make money while others were drawn to use IDU out of boredom, curiosity, or an attempt to be part of a social circle. As graduates from universities and technical colleges, young adults often had to travel to larger cities to find employment. When they first arrived they would try to find a social circle and a place to belong. This was how many people were introduced to people who inject drugs, according to IDUs and substance abuse treatment providers. They would then introduce their friends to IDU upon returning home and consequently form small IDU communities. Furthermore, the consumption of alcohol is a very important part of the Ukrainian culture. With the economic situation under strain, people turned to alcohol to escape the harsh realities of everyday life. IDU is slowly becoming the mechanism of abuse among young people who wished to do the same. Though information collected through different sources did not contradict this observation, more research is needed to confirm that there is indeed an association between alcohol use and illicit drug use.

Another important factor that was indicated as a possible facilitator of drug use, and one that should also be explored through further research, is the changing social environment among school-aged children. As the economy began to crumble, many parents were forced to take on additional jobs to compensate for low income. Their children were under less supervision, and some community representatives speculate that they became more vulnerable to exploring drugs. Extracurricular activities such as sports and various special interest clubs were free-of-charge during the Soviet era. Following the fall of the Soviet Union, however, local schools no longer had the necessary financial resources to sponsor extracurricular activities, and thus children were left with more free time and less to do. These are factors that may play a role in the increasing IDU epidemic. However, more research is needed in this area.

While young people from the Vinnitsya community were exposed to various forms of pro-drug messages (including the cultural acceptance of alcohol abuse) on practically a daily basis, the effort to counteract the influence of these messages by informing the public about the dangerous consequences of substance abuse was minimal. Aside from the recent implementation of school-based anti-drug programs and a special radio program that reaches out to the Vinnitsya city residents, there was very little evidence of anti-drug propaganda (i.e. in terms of social marketing and regulation). Furthermore, not much was said about the association between alcohol abuse and HIV/AIDS. This was especially true in the rural areas, where the information was needed the most.

### Drug use prevention and treatment

The promotion of anti-drug messages in schools tended to be the most favored primary prevention approach toward controlling the spread of IDU in this area. The RND has been active in launching several school-based anti-drug prevention programs in all 40 local schools in Vinnitsya, as well as schools in the surrounding areas. Most schools and teachers were surprisingly open to this type of intervention. Students were also quite open and interested in the topics presented and actively asked and answered questions. These programs, however, did not appear to be carried out to their fullest potential. They were not theory-based or standardized in their delivery. The focus of the sessions was primarily on IDU and excluded more common substances such as tobacco and alcohol. The programs were mostly didactic with no experiential components. Teaching materials and information were not always age appropriate, and there were no hand-out materials or resources to obtain help or further information about drug abuse. Furthermore, the programs did not have a built-in evaluation system.

Another school-based primary prevention program implemented by a local NGO, titled Project Hope, was modeled after a project that started in the United States and has been implemented in many areas around the world – including Moscow and three Ukrainian cities (Kharkiv, Odessa, and Kremenchoug). The goal of the program was to teach children in 1^st^–4^th ^grades self-efficacy skills (i.e. how to listen, hear, speak, understand and act with the attitude of "I'm free" and "I know how to live") through fairy tales, games, and various fun activities. Teachers also were provided with guides on how to conduct interactive sessions and ideas on how to get parents involved. Older children were encouraged to participate in a voluntary Children's Club, in which children taught each other about the negative consequences of drug, alcohol and tobacco use and sexual activity, as well as sexually transmitted diseases. This program, however, was being implemented at only one school in the Vinnitsya area, and no information on its efficacy has been presented.

The local media also were involved in primary prevention. The public radio aired a special program on drugs, during which various substance-abuse specialists talked about the consequences of drug abuse and how to deal with addiction. The program also included individuals who had personal experience with substance abuse and were willing to share their story with others. The goal of the program was to provide people with the necessary information to encourage young people not to take drugs or encourage individuals who use drugs to seek treatment. The local newspaper also has created a special section with information about drug use (i.e. general information, consequences, and resource contact information). Most of this information, however, was limited to individuals who live in Vinnitsya and did not reach the rural areas, where the information also was needed.

In the area of tertiary IDU prevention, a local NGO has been carrying out a needle/syringe exchange program (NEP/SEP) at various sites in the Vinnitsya area over the past three years. There were two NEP/SEP sites in Vinnitsya, as well as sites in Kalynovka, Ladyzhyn, Zhmerynka, and Haysin, with one being planned for Kaziatin. IDUs in the area were able to exchange used needles and syringes for new, sterile needles and syringes at no cost. The goal of the program was to reduce the potential harm of IDU by reaching out to individuals who put themselves at risk for physical injuries, nerve damage, and infection with sexually transmitted diseases and various blood-borne viruses including HIV, Hepatitis B and Hepatitis C. Educational booklets distributed included information on safer injection practices (to avoid serious injuries such as nerve damage), safer sex practices, and information on where to go for treatment.

Though the authorities have supported harm reduction programs such as this, needle exchange was not yet fully accepted within the community. There was still some doubt as to the effectiveness of such a program, and there was even some suspicion that NEP/SEP served as a place to purchase drugs. According to the social workers from the Vyshenka District NEP, the local police had been suspicious that the social workers were involved in the drug trade. The police had been known to send in undercover agents, for instance, to see whether they could purchase drugs or poppy straws from the social workers. The program was advertised through "word of mouth" due to the stigma placed on IDUs. There tended to be a lack of coordination between the NGO and other organizations in the community, all of which would help to make the program more effective. No empirical information on the effectiveness of this program has been presented to the research or local community over the three years of program operation.

The RND (a 160-bed inpatient, government supported hospital) is the only facility for the diagnostics and medical treatment of alcohol and drug related disorders in Vinnitsya. Located in the south Leninsky district, it is staffed by medical doctors, psychologists, social workers, and nurses. It also carries out the expert assessment of substance use disorders for local road police and the criminal justice system and provides educational lectures to local and rural elementary schools. There were more 24 thousand persons "registered" as patients of the RND. The patient population was primarily male and approximately 60 percent from the city of Vinnitsya and 40 percent from rural areas. Most patients were being treated for alcohol-related disorders. Only 10 percent were opiate IDUs, despite the increasing demand for treatment of this problem. Treatment was organized into three stages: medical, psychotherapy, and rehabilitation. Primary emphasis was placed on medical treatment.

Though the RND is a public health facility, the government covers only a small portion of the total cost of treatment, which barely covers staff salaries. Treatment in public institutions is considered free, which means that patients do not pay any hospital or physician charges. However, since the breakdown of the Soviet system, the state budget does not make provisions for medication. Consequently, patients were responsible for covering the cost of medicines and various medical supplies, which usually added up to about 150 grivnas ($28) for the minimum length of stay – an amount that exceeded some people's monthly salary. The RND provided treatment primarily for the patients' physical dependency (medical detoxification) with very little aftercare, and it was plausibly reported that many patients have short remission times and return to alcohol or drug abuse just a few months following the completion of the treatment. Each patient is recommended to follow up with a psychologist or a regional narcologist after discharge; however no monitoring system is in place, which prevents valid outcome assessment. According to the treatment providers' personal experience, very few patients do see their psychologist or narcologist before the next full-blown relapse. Factors that may hinder the effectiveness of the treatment include: high cost and short length of treatment, lack of evidenced-based interventions, lack of outpatient treatment and drug use monitoring, and absence of narcotic substitution medication therapies. There are essentially no eligibility criteria for admission; any resident of Vinnitsya oblast could enter the RND if diagnosed with a substance use disorder. High costs of medication and little faith in the efficacy of available treatment among potential patients further decrease the attractiveness of drug treatment in Vinnitsya.

There was a great amount of prejudice toward IDUs both within the community and among medical professionals that presented another barrier to the effective treatment of IDUs. Interviews with medical practitioners revealed discriminatory attitudes toward IDUs. They were perceived to be criminals and/or individuals who lack moral values. Furthermore, IDUs were not the most "liked" patients, even at the RND, where patients with alcohol dependency were said to get preferential treatment. Alcohol dependency may be more acceptable due to the strong cultural role of alcohol in this society. Some medical professionals viewed IDUs as "hopeless cases" or as individuals who are "impossible to treat." It is commonly believed that IDUs generally were not willing to change their lifestyle and came for treatment only in order to lower their tolerance level (as it becomes too expensive for them to inject) or avoid a prison sentence. Furthermore, some health professionals explained that IDUs are so mentally disturbed (due to brain damage) that they are no longer receptive to any treatment. Direct observations at the treatment center revealed that it was not unlikely to see a physician on duty refuse treatment to HIV positive IDUs and send them to another facility (i.e. an infectious disease hospital). The director and staff of the RND, however, were highly compassionate and motivated to improve the state of treatment services for alcohol and drug related disorders at the RND.

## Recommendations

A response designed to positively impact mortality and morbidity associated with HIV and IDU in this part of the world must be swift and comprehensive. Given that Ukraine has sparse resources to be devoted to this problem, action recommendations should be prioritized, realistic, and initially targeted to persons in greatest need. The following action recommendations are prioritized by the following categories: First priority: Voluntary Counseling and Testing; Second Priority: Prevention and Education; and Third Priority: Harm Reduction and Treatment. They are provided in this sequence based on what response can realistically be implemented first with limited additional resources and can make the greatest immediate impact. The persons at greatest risk, HIV positive persons and IDUs, should be attended to first.

The existing ICOHRTA has a four-year history of training health care providers, medical students, and drug addiction specialists and has a relationship with existing agencies capable of responding to the HIV and IDU epidemic. As such, we recommend using the resources and staff of ICOHRTA for initiating and sustaining the implementation of the following priorities. IROHRTA resources and staff can link in-country stakeholders with educational materials, risk assessments, skill building protocols, and evidence-based prevention and intervention protocols, training. They also can offer assistance with writing grants to procure funds for HIV testing supplies, condoms, bleach kits, educational materials, research, and salary support for prevention staff.

### First Priority: Voluntary Counseling and Testing

Voluntary counseling and testing for HIV (VCT) is often used as the first step in addressing HIV transmission and prevention. Most people who have HIV do not know they have it. Furthermore, many people at risk are afraid to be tested because of the stigma associated with IDU and HIV and fear of testing positive. One way to address the AIDS epidemic is to give people an opportunity to know their HIV status so that they can take precautions to avoid further spread and seek treatment if they are infected [25,26]. Appropriate agencies should be identified by ICOHRTA investigators for training and implementation of VCT.

Identification of those in need of help through effective outreach, motivational enhancement, risk assessment, and VCT must be the first priority to make an immediate impact on those at greatest risk in Vinnitsya. Vinnitsya has various locations appropriate for VCT sites. For example, the RND, the Infectious Disease Hospital, PMU, the STEPS program (an outpatient drug and alcohol addiction treatment center), and the NEP NGO are existing organizations that could offer VCT services. Training of agency staff is recommended in effective peer outreach strategies, risk assessment, motivational enhancement, and VCT protocols. Immediate diversion of agency funds and applications for small grants are recommended to fund the purchasing of HIV test kits. Successful outreach and VCT will immediately break down the first barrier to assessing the problem through anonymous testing, epidemiological prevalence surveys, and provision of prevention, education, and treatment to those most in need.

### Second Priority: Prevention and Education

As a result of effective outreach and VCT, four high risk populations will emerge, in order of severity: HIV positive IDUs, HIV positive non-IDUs, HIV negative IDUs, and HIV negative non-IDUs. The second priority is the prevention of HIV transmission among high risk populations through education, skills development, and distribution of free condoms with support from the ICOHRTA. Secondary prevention or the prevention of the spread of HIV by persons who are HIV positive IDUs or HIV non-IDUs should be given the greatest attention. Since only persons with HIV can spread HIV, reducing risk behaviors among persons who are HIV positive can make the most immediate impact on transmission rates.

HIV positive IDUs should be targeted first for secondary prevention by VCT providers via education about their risk of transmitting HIV to others and how to prevent transmission through abstinence or reduce it through condom use. They should first have access to free condoms and be trained in correct condom use and then interpersonal sexual negotiation skills to practice safer sex or abstinence. HIV positive IDUs should then be educated about the HIV transmission risks associated with IDU and the dangers associated with sharing needles, syringes, paraphernalia, or drug solutions. Drug and alcohol use in general reduces the practice of safer sex and drug use behaviors, but IDU is the most risky behavior due to the opportunity to spread the virus by sharing needles, syringes or other contaminated drug use paraphernalia. Therefore, training in needle and syringe cleaning with bleach should be given high priority.

Persons who are HIV positive may be suffering from despair and depression and be less motivated than high risk populations to practice safer sex or IDU behaviors. It is recommended that VCT prevention staff be adequately trained in the areas of coping with HIV diagnosis, apathy, depression, and even suicidal ideation. Finally, prevention staff should work on identification and notification of sexual and IDU partners. HIV positive IDUs represent a significant risk to their sex and drug use partners. HIV positive IDUs should be encouraged to inform partners of their HIV status so the partners can have the opportunity to practice HIV prevention themselves.

The next two high risk populations in order of severity are HIV negative IDUs and HIV negative non-IDUs. They should be targeted next for prevention and education. Many of the prevention strategies proposed above for HIV positive persons apply to these groups. HIV negative IDUs should be given the first priority because of their risky practice of IDU. They should be provided with the abovementioned programs to prevent drug use prevention, teach sexual negotiation skills and provide condoms. They also should be encouraged to return for re-testing after three months. Finally, HIV negative non-IDUs are the next target population. This group should be assessed for unsafe sex behaviors and non-IDU risky drug and or alcohol use. They should be provided with condoms, HIV prevention education, risk reduction skills, and information about the use of drugs and alcohol and practicing unsafe sex.

### Third Priority: Harm Reduction and Treatment

Once high risk populations are identified and tested for HIV, and prevention and education have been implemented, attention should be given to reducing harm from existing risky behaviors and enhancing opportunities for treatment. The philosophy of harm reduction, as opposed to abstinence, should guide the initial delivery of tertiary prevention. While abstinence from both sex and IDU behaviors is the only safe way to prevent HIV transmission, this advice is often met with resistance, takes a long time to achieve, and is not realistic for many persons. The most commonly practiced harm reduction method to limit the spread of HIV among IDUs is implemention of NEP/SEP. Vinnitsya has had an NEP funded by a NGO for the past several years. This NEP reaches out to the community from two street sites, offering clean needle exchange and alcohol swabs. The concept of NEPs in Vinnitsya is not fully accepted by the police, and the NEP sites are regularly monitored for any illegal practices. It is recommended that the ICOHRTA investigators meet with the leader of this NGO and discuss ideas of offering free condoms and prevention literature, making referrals for treatment, site expansion, and additional funding. Strategies should be discussed about how to link the NEP with treatment providers in the community to refer persons needing and motivated to seek out drug addiction treatment or medical services related to IDU or HIV/AIDS.

Treatment for IDU and HIV/AIDS is limited in Vinnitsya. However, since the beginning of the ICOHRTA approximately four years ago, training, research opportunities, and grant funding for treatment of both IDU and HIV/AIDS has increased. Assessment of drug addiction treatment model preferences was assessed among providers and patients of the RND in preparation for the technology transport of behavioral, HIV prevention, motivational enhancement, and relapse prevention interventions [21]. The National Institute of Drug Abuse (NIDA) is funding this transport of interventions for IDU and HIV risk under the leadership of the second author through training in evidenced-based psycho-social and behavioral treatments, increased use of family support, and ultimately sustaining a raised standard of care at the RND. Finally, there is an increase in antiretroviral treatment of HIV/AIDS in Vinnitsya as a result of grants to the fourth author. It is recommended that the ICOHRTA continue to support such efforts and increase in-country investigators' independence in conducting community-based developmental research that will immediately raise the standard of care for IDU and HIV/AIDS, encourage further innovation, and ultimately have a positive effect on reducing the prevalence of HIV/AIDS.

## Conclusion

It has been stated that in order to control the HIV/AIDS epidemic, one must first understand the cultural, political, economic, and religious context in which populations, individuals, and their behaviors are situated [22]. An increase in IDU in Ukraine within the last decade, for instance, may be explained by complex factors such as the country's economic crisis, rapid social change, and increased poverty and unemployment [7]. A high number of out-of-treatment IDUs within Ukraine's society may be explained by the lack of effective substance abuse treatment, limited number of HIV prevention programs for IDU, and/or stigma toward individuals who take part in substance abuse [14]. The findings of this study support these theories with respect to HIV/AIDS and IDU in the city of Vinnitsya, Ukraine.

Though the Vinnitsya Oblast is not the most severely affected region in terms of HIV infection, the assessment of the current state of the HIV/AIDS in this area of the country does shed some light on what is going on there. First, we have learned that there is a great shortage of resources for testing of HIV/AIDS (especially in the less populated areas) and low HIV/AIDS awareness in the general population. It is therefore possible that the statistics from these areas may be inadequate due to underreporting. Thus, there may not be such a large difference between the heavily affected regions (i.e. Odessa, Mykolaiv, Dnipropetrovs'k, and Donets'k) and other regions of the country. The most heavily infected regions may simply have better reporting systems and resources for HIV testing.

Second, while the statistics for Ukraine as a whole seem to indicate that there is a shift toward heterosexual transmission of HIV, a great majority of the HIV infections reported prior to 2004 in the Vinnitsya region were among IDUs. It seemed plausible, that the HIV/AIDS epidemic in the Vinnitsya region was a few years behind. In fact, there were no officially registered HIV infections in this region until the year 1996, and the current distribution of HIV infections by risk group actually resembles the national statistics for that same year. Unexpectedly, the majority of newly registered HIV cases in Vinnitsya in 2004 were attributed to heterosexual transmission (54.1%). It can be explained either by a true shift in the epidemic dynamics or by improved surveillance after the establishment of the Regional AIDS Centre; however, there are no sufficient data to document either explanation. In any case given that the great bulk of existing HIV cases are among IDUs it is reasonable to conclude that high, significant problems lie ahead without enhanced prevention and treatment efforts.

There also is a shift in the nature of drug use and the demographics of individuals who abuse drugs in this part of the country. Currently, individuals who abuse drugs tend to be younger, and the drugs tend to be more potent and riskier than they were when Ukraine was a part of the Soviet Union. Drug abuse appears to start with the use of alcohol and smoking of cannabis among children as young as 10 years of age, which may help to explain the earlier onset of IDU. Konoplya, a strong cannabis-based substance that was not as common among teenagers during the Soviet era (perhaps due to a stricter regulation at the borders), may serve as a gateway drug, leading the way to experimentation with more risky drugs (i.e. homemade stimulants and opiates). Because many IDUs make the opiate solution themselves in non-sterile home-laboratories, they have control over its strength; face a greater chance of contaminating the solution (i.e. by using unclean works and solvents); and present great risks of HIV transmission through needle and drug sharing. Prevention and treatment efforts have been attempted with limited success due to inadequate resources, training, and the burgeoning IDU problem in the area. Avenues for change in the drug addiction prevention and treatment fields are open due to caring and motivated health care providers and public health officials. In order to control the HIV/AIDS and IDU epidemics, one needs to consider a great deal of factors that fuel these epidemics. Changes may have to be made at the levels of individuals, services, communities, environments, and policies. Consequently, it will take a collective effort to make significant progress.

## List of Abbreviations Used

ICOHRTA – International Clinical, Operational and Health Services Research and Training Award

IDU – Injection Drug Use

IDUs – Injection Drug Users

IDU-RAR – Rapid Assessment and Response in Injection Drug Use

NEP/SEP – Needle/Syringe Exchange Program

NGO – Non-governmental Organization

PMU – Vinnitsya National Pirogov Medical University

RND – Regional Narcological Dispensary

UAB – University of Alabama at Birmingham

WHO – World Health Organization

## Competing interests

The author(s) declare that they have no competing interests.

## Authors' contributions

Katerina Barcal, MPH conceived of the study and its design, the collection of data, organizing the data for qualitative analysis, drafting the article and making final edits, and giving final approval for publication.

Joseph E. Schumacher, PhD mentored Ms. Barcal in the study conceptualization, design, data analysis, manuscript preparation, giving final approval for publication.

Kostyantyn Dumchev, MD, MPH participated in the design and methodology of the study, collection of the data, organization of the data, editing the manuscript, and giving final approval for publication.

Larisa Vasiliyevna Moroz, MD, PhD participated in the acquisition and interpretation of the data, editing the manuscript, and giving final approval for publication.
